# Supplementary material for: Effects of Quantum Noise and Source Blurring on Dark-Field Signal Retrieval in X-ray Speckle-Based Imaging
Source: ArXiv. 2026 Aug 25:arXiv:2608.24806v1. Preprint. [Version 1] (PMC13532704)
Supplement: Supplementary file 1 [file NIHPP2608.24806v1-supplement-1.pdf]

Supplementary Material

S1 Simulation Methodology

To further elucidate the findings reported in the main text—specifically the observation that the smallest focal spot size yielded the highest MSE while maintaining a PCC comparable to the 20 μm source—we conducted a numerical analysis using simulated jittered speckle patterns. This supplementary study aims to characterize the interaction between a stochastic scattering surface, focal spot size, and detector-level discretization under mechanical instability.

S1.1 Stochastic Surface Modeling

To simulate a realistic scattering medium, we employ a multi-scale spatial correlation model. The process begins with the generation of a high-resolution 2D array of uncorrelated white noise, denoted as $I_{raw}(x, y)$.

To introduce physical correlation lengths representing different grain sizes, we apply a series of Gaussian filters to the raw noise. The multi-scale base pattern $P(x, y)$ is defined as the summation of three distinct spatial regimes:

$$P(x,y) = \sum_{i \in \{2,6,15\}} G(x, y; \sigma_i) * I_{raw}(x, y) \tag{S1}$$

where $G(x, y; \sigma_i)$ represents a Gaussian kernel with standard deviation σ_i. These values correspond to fine microscopic grit ($\sigma = 2$), nominal macroscopic grains ($\sigma = 6$), and large-scale structural clustering ($\sigma = 15$).

S1.2 Focal Spot Blurring

The optical system is modeled as a low-pass filter defined by the focal spot size. The effective optical field I_{opt} is obtained by convolving the base pattern with a Gaussian point spread function (PSF):

$$I_{opt} = P(x, y) * G(x, y; \sigma_{spot}) \tag{S2}$$

Following experimental approximations, the standard deviation of the blur kernel is related to the spot size by $\sigma_{spot} = \text{spot\_size} \times \frac{3}{7}$.

S1.3 Detector Discretization and Jitter

The high-resolution optical field is discretized to match the detector pixel pitch through a spatial integration process. For an effective pixel size D, the detected intensity I_{det} is calculated by averaging the high-resolution grid within a $D \times D$ window.

Mechanical jitter is introduced as a discrete spatial shift Δx applied to the high-resolution field prior to the downsampling process. This allows for the simulation of sub-pixel vibrations, where $\Delta x < D$.

S2 Parameter Selection and Experimental Alignment

The simulation parameters were specifically selected to maintain parity with the experimental configuration:

- **Effective Pixel Size (17 μm):** Set to match the detector pitch used in high-resolution data acquisition.
- **Focal Spot Sweep (5 μm to 60 μm):** Selected to characterize the transition from the diffraction-limited regime to the blur-dominated regime.
- **Mechanical Jitter (2 μm):** Introduced to simulate typical sub-pixel mechanical instabilities observed in the experimental setup.
- **Additive Noise ($\sigma \in [0, 0.01]$):** Additive White Gaussian Noise (AWGN) was incorporated to evaluate the robustness of correlation metrics against varying signal-to-noise ratios (SNR).

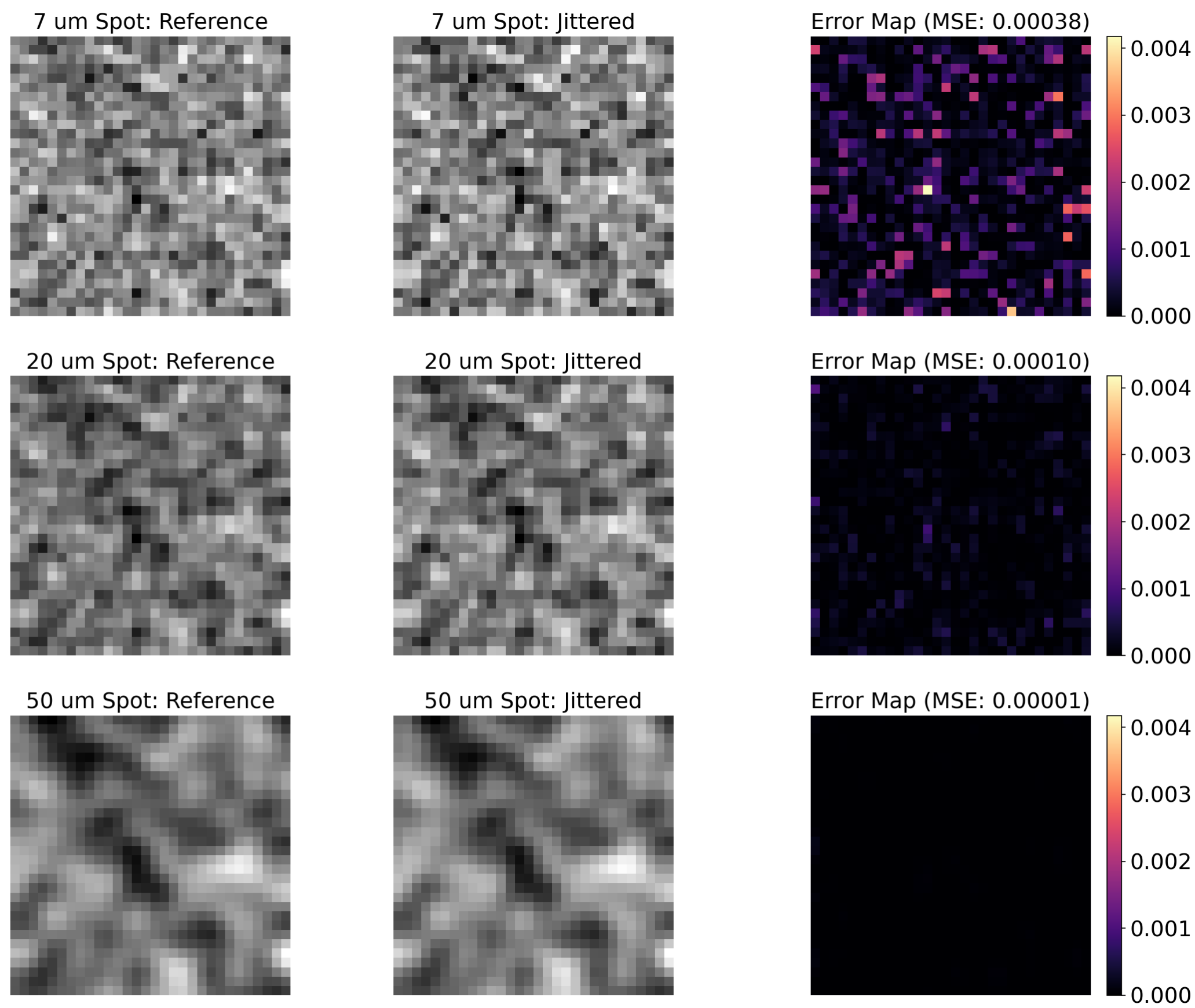

Figure S1: Representative simulated speckle patterns for various focal spot sizes ($7, 20$, and $50\ \mu$m). The columns from left to right display the original speckle patterns, their corresponding jittered counterparts, and the resulting MSE maps, respectively.

S3 Results

Figure S1 (left column) presents the simulated speckle patterns for focal spot sizes of $7, 20$, and $50\ \mu$m, alongside their jittered counterparts (middle column) and corresponding MSE maps (right column). To provide a more comprehensive characterization, the MSE and PCC were quantified as a function of focal spot size, as shown in Fig. S2 and S3, respectively.

The simulation results clearly demonstrate that the $7\ \mu$m focal spot yields the highest MSE, with values decreasing as the source size increases. Furthermore, the PCC for the $7\ \mu$m source remains comparable to that of the $20\ \mu$m source, followed by a decline as the source size increases further. These numerical findings are in excellent agreement with the experimental observations presented in Fig. 4(c) and (d) of the main text, thereby validating the robustness of our retrieval analysis.

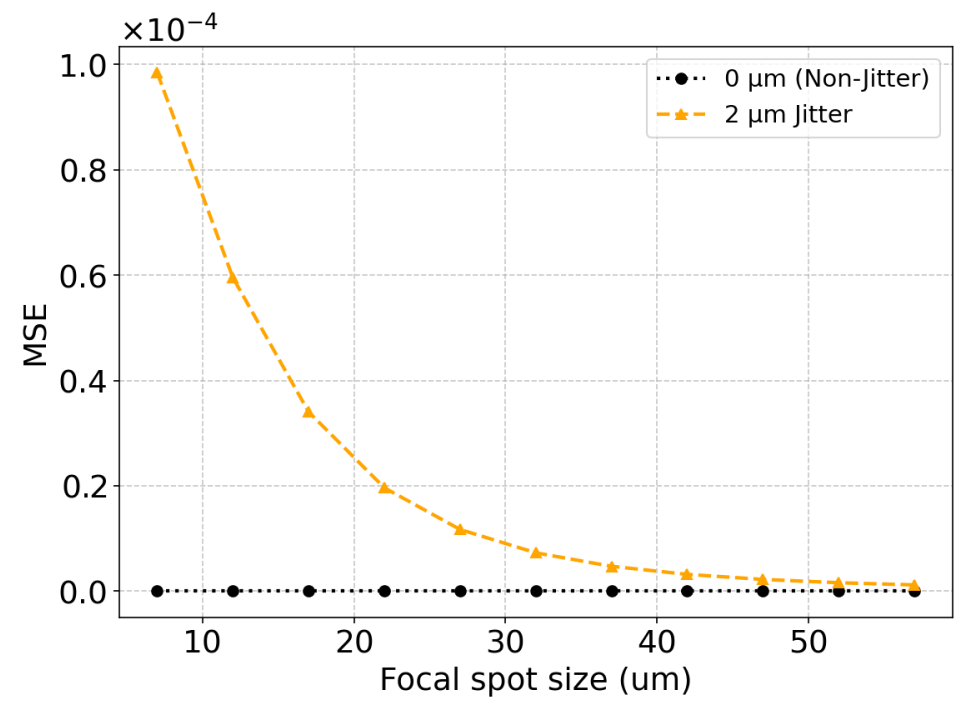

Figure S2: Mean Squared Error (MSE) as a function of focal spot size.

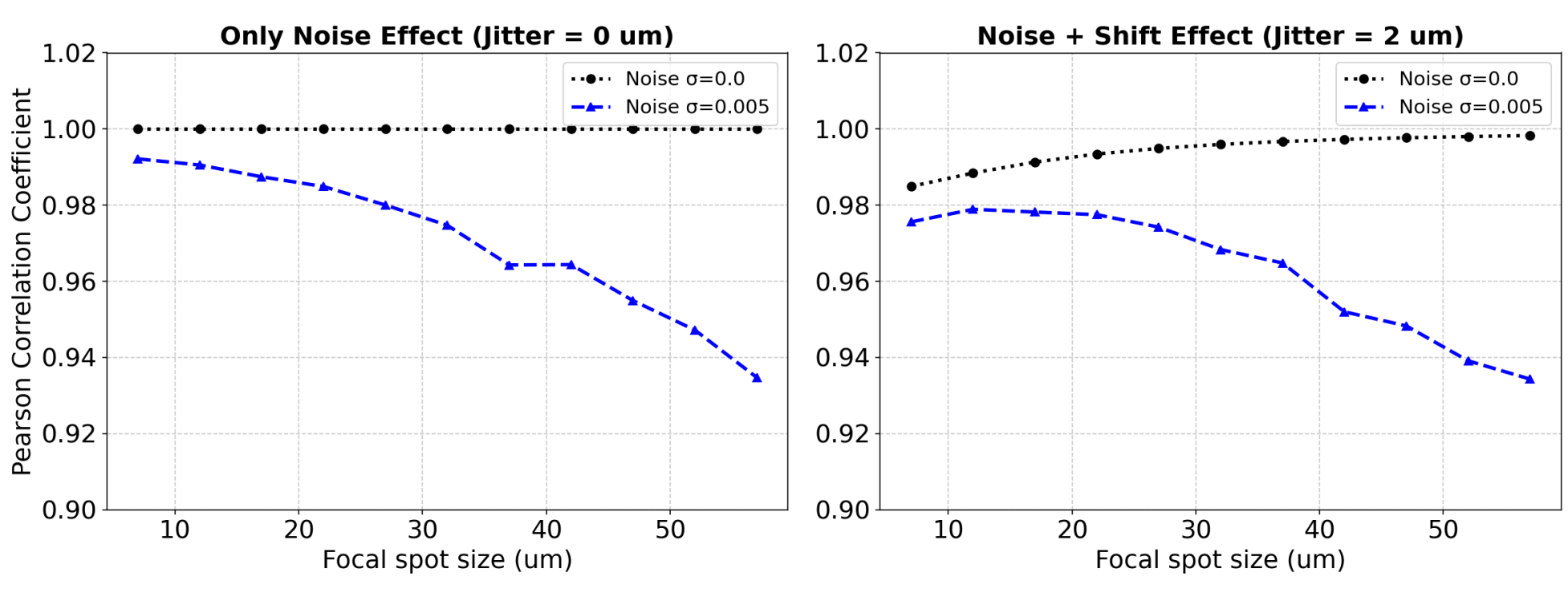

Figure S3: Pearson Correlation Coefficient (PCC) as a function of focal spot size.
